# Cystatin M loss is associated with the losses of estrogen receptor, progesterone receptor, and HER4 in invasive breast cancer

**DOI:** 10.1186/bcr2783

**Published:** 2010-11-23

**Authors:** Eunkyung Ko, Seong-Eun Park, Eun Yoon Cho, Yujin Kim, Jung-Ah Hwang, Yeon-Su Lee, Seok Jin Nam, Saik Bang, Joobae Park, Duk-Hwan Kim

**Affiliations:** 1 Department of Molecular Cell Biology, Sungkyunkwan University School of Medicine, 300 Cheoncheon-dong, Jangan-gu, Suwon, Gyeonggi-do 440-746, Korea; 2 Department of Pathology, Samsung Medical Center, Sungkyunkwan University School of Medicine, 50 Irwon-dong, Gangnam-gu, Seoul 135-710, Korea; 3 Functional Genomics Branch, National Cancer Center, 111 Jungbalsan-ro, Ilsandong-gu, Goyang-si, Gyeonggi-do 410-769, Korea; 4 Department of Surgery, Samsung Medical Center, Sungkyunkwan University School of Medicine, 50 Irwon-dong, Gangnam-gu, Seoul 135-710, Korea

## Abstract

**Introduction:**

This study was aimed at understanding the clinicopathological significance of cystatin M loss, and investigating possible factors responsible for cystatin M loss in breast cancer.

**Methods:**

The expression of estrogen receptor (ER), progesterone receptor (PR), HER2, HER4, and cystatin M was retrospectively analyzed using immunohistochemistry in 117 patients with ductal carcinoma *in situ *(DCIS) and in 175 patients with invasive breast cancer (IBC). The methylation status of *CST6 *gene encoding cystatin M was evaluated using methylation-specific polymerase chain reaction (PCR) in formalin-fixed paraffin-embedded tissues from 292 participants and using pyrosequencing in fresh-frozen tumor and matched normal tissues from 51 IBC patients.

**Results:**

Cystatin M loss was found in 9 (8%) of 117 patients with DCIS and in 99 (57%) of 175 with invasive breast cancer (IBC) (*P *< 0.0001). Cystatin M loss was found in 58 (57%) of 101 HER2-negative IBCs and in 41 (55%) of 74 HER2-positive IBCs, and this difference was not statistically significant (*P *= 0.97). However, cystatin M loss was significantly associated with the loss of ER (*P *= 0.01), PR (*P *= 0.002), and HER4 (*P *= 0.003) in IBCs. Cystatin M loss occurred in 34 (76%) of the 45 HER4-negative IBCs and in 65 (50%) of the 130 HER4-positive IBCs. Multivariate analysis showed that cystatin M loss occurred at a 3.57 times (95% CI = 1.28 to 9.98; *P *= 0.01) higher prevalence in the triple-negative IBCs of ER, PR, and HER4 than in other subtypes, after adjusting for age. The quantity of *CST6 *methylation was associated with ER loss (*P *= 0.0002) in IBCs but not with the loss of PR (*P *= 0.64) or HER4 (*P *= 0.87).

**Conclusions:**

The present study suggests that cystatin M loss may be associated with the losses of ER, PR, and HER4 in IBC.

## Introduction

Ductal carcinoma *in situ *(DCIS) of the breast is the most common type of noninvasive breast cancer in women and accounts for 20 to 30% of breast cancer detected by screening mammography [1,2]. Abnormal cells in DCIS are confined to the lactiferous ducts in the breast and do not spread into the surrounding stroma. However, further changes in cells comprising DCIS lesions result in the destruction of the basement membrane that surrounds the duct and in the spread of tumor cells into the surrounding tissue. Lysosomal cysteine proteases are involved in the degradation of components of the extracellular matrix *in vitro*, and increased activity of these proteases leads to the destruction of the extracellular glycoprotein scaffolds that maintain tissue architecture, thus facilitating invasion of cancer cells beyond the basement membrane.

Cystatin M is a candidate tumor suppressor that functions as a physiological inhibitor of lysosomal cysteine proteases. Cystatin M is abundantly expressed in normal and premalignant breast epithelium, but its expression has been reported to be diminished or lost in breast cancers [3-7]. The loss of cystatin M expression is associated with the progression of primary tumors to a metastatic phenotype [3,4,7]. Furthermore, exogenous expression of recombinant cystatin M results in the suppression of cell proliferation, migration, and matrix invasion *in vitro *[8]. The *CST6 *gene encoding cystatin M contains a large CpG island that spans the proximal promoter and exon 1, encompassing the transcription start site. Several groups have reported DNA methylation-dependent silencing of *CST6 *gene in breast cancer cell lines and primary invasive ductal carcinomas, but the upstream initiators that direct this process have not been elucidated [5,6].

Recently, Leu *et al*. [9] reported that disruption of the estrogen receptor ERα in breast cancer cells resulted in DNA methylation of ERα target genes. In addition, a number of studies have reported a unique relationship between ER and HER4 in breast cancer [10-14]. Zhu *et al*. [14] reported that ER and HER4 can target estrogen-inducible gene promoters such as stromal cell-derived factor 1 (SDF-1), a putative key player of the matrix remodeling. Based on these reports, we hypothesized that cystatin M may be a downstream target of ER and/or HER4 and that *CST6 *methylation may be influenced by the alteration of ER and/or HER4. To investigate the clinicopathological significance of cystatin M loss and to identify possible factors associated with cystatin M loss in breast cancer, we analyzed the expression status of five proteins (ER, PR, HER2, HER4, and cystatin M) and the hypermethylation of *CST6 *gene in a total of 292 breast cancer patients.

## Materials and methods

### Study population

A total of 117 DCIS and 175 IBC patients participated in this study. Pure DCIS cases were included in this study, and DCIS lesions associated with invasive breast cancer were excluded. All specimens were obtained from patients who underwent surgical resection for DCIS and IBC between May 2001 and July 2006 at the Samsung Medical Center in Seoul, Korea. Written informed consent for the use of the surgically resected tumor tissues was provided by all of the DCIS and IBC patients prior to operation. Paired samples of primary breast cancer and matched noncancerous normal tissues were obtained from each patient. Surgically removed tissues were immediately snap-frozen in liquid nitrogen and stored at -80°C until use. All procedures used in this study were approved by the Institutional Review Board at the Samsung Medical Center. Information regarding demographics and lifestyle factors was obtained from an interviewer-administered questionnaire. Patient age ranged from 28 to 72 years for DCISs and from 25 to 83 years for IBCs. The pathologic stage of the IBC was classified according to the American Joint Committee on Cancer (AJCC) TNM criteria (sixth edition).

### Tissue microarrays and immunohistochemistry

**Figure 1:**
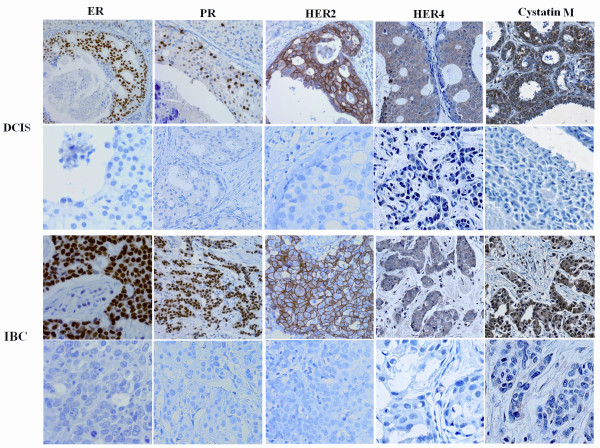
**Representative immunohistochemical statinings of ER, PR, HER2, HER4, and cystatin M in DCIS and IBC lesions**. The positive (upper row) and negative (lower row) immunostainings for cystatin M, ER, PR, HER2, and HER4 are shown in DCISs and IBCs. Most cells in the upper row of DCIS and IBC lesions show strong nuclear reactivity for ER and PR, strong membrane reactivity for HER2, and strong cytoplasmic staining for HER4 and cystatin M. Magnification × 400.

Tissue microarrays (TMAs) of DCISs and IBCs were prepared as described previously [15]. Briefly, tissue sections of four-micrometer-thickness were taken from the TMA blocks for immunohistochemistry and deparaffinized in xylene and rehydrated through a series of alcohols. The rehydrated sections were treated with 3% hydrogen peroxide in methanol for 10 minutes to block endogenous peroxidase. To unmask the antigens, sections were microwaved in citrate buffer (pH 6) for a total of 15 minutes. Sections were incubated with a primary antibody overnight at room temperature after blocking nonspecific proteins with 2% dried milk in phosphate-buffered saline (PBS) for 30 minutes and 5% goat serum in PBS for 1 h. The antibodies used were ER (clone 6F11, Novocastra, Vision Systems Inc., Norwell, MA, USA, diluted at 1:400), PR (clone 16, Novocastra, diluted at 1:800), C-erb2 (clone CB11, Novocastra, diluted at 1:400), C-erb4 (clone HFR-1, Neomarkers, Fremont, CA, USA, diluted at 1:30), and cystatin M (clone 211515, R&D Systems, Minneapolis, MN, USA, diluted at 1:20). Detection of immunoreactivity by each antibody was performed by the Vectastain Elite ABC reagent (Vector Laboratories, Burlingame, CA, USA) and 3.3'-diaminobenzidine tetrahydrochloride was used as a chromogen. All sections were counterstained with Mayer's hematoxylin, and negative controls were included in each staining sequence and obtained by omitting the primary antibodies. Cytokeratin 8/18 (Invitrogen, Carlsbad, CA, USA; Cat. No. 18-0213) was used as a positive control of staining to verify that tissues were stained. Representative stainings of ER, PR, HER2, HER4 and cystatin M expression in DCISs and IBCs are shown in Figure 1.

### Interpretation of immunohistochemistry

Cutoff values for positive or negative expression were determined considering widely accepted criteria among previously reported studies and expression patterns in our normal tissues (data not shown). The positivity of expression was assessed semiquantitatively by evaluating the percentage of positive staining cells and/or staining intensity. The expression of ER and PR was determined according to the Allred score. If the sum of both scores is more than three, the expression of ER and PR was considered positive. HER2 expression was considered as positive when moderate to strong membrane immunoreactivity was observed in more than 10% of tumor cells. The membrane or nuclear staining of HER4 was not considered in the final data analysis due to low frequency (< 5%) of the staining in IBCs. Only cytoplasmic reactivity was considered evidence of HER4 as well as cystatin M expression. An immunoreactive score (IS) of 0 to 7 for HER4 or cystatin M expression was obtained by adding the intensity score (0, none; 1, weak; 2, moderate; 3, strong) and a proportion score of positive staining tumor cells (0, absent; 1, 0 to 10%; 2, 10 to 50%; 3, 50 to 80%; 4, >80%). If the IS was less than two, the tumor was considered to exhibit loss of HER4 or cystatin M expression.

### Methylation-specific PCR (MSP)

Genomic DNA was extracted from fresh-frozen tissues and formalin-fixed paraffin-embedded tissues using a DNeasy Tissue Kit (Qiagen, Valencia, CA, USA) according to the manufacturer's instructions. The methylation status of the *CST6 *gene in the formalin-fixed paraffin-embedded tissues was determined using methylation-specific PCR (MSP), as described by Herman *et al*. [16]. The MSP primers specific for the methylated *CST6 *gene were 5'-GTTTTTTGAATTTCGTAGGATTTC-3' (sense) and 5'- AACTTTTACCCGCTAAACCG-3' (antisense). The primers for the unmethylated *CST6 *gene were 5'-GGTTTTTTGAATTTTGTAGGATTTT-3' (sense) and 5'- CAACTTTTACCCACTAAACCACC -3' (antisense). The PCR conditions for DNA amplification consisted of 1 cycle at 95°C for 7 minutes; 35 cycles at 94°C for 30 s, 65°C (for M primer set) or 63°C (for U primer set) for 1 minute, 72°C for 1 minute; and final extension at 72°C for 10 minutes. DNA from the peripheral blood lymphocytes of healthy subjects was used as a negative control for the methylation-specific assays. Lymphocyte DNA from healthy volunteers was treated with *Sss*1 methyltransferase (New England BioLabs, Beverly, MA, USA), and then with sodium bisulfite. The resulting product was used as a positive control for methylated alleles. Bisulfite-modified DNA from normal lymphocytes was used as a positive control for unmethylated alleles, and the unconverted DNA from normal lymphocytes was used as a negative control for the methylated alleles.

### Pyrosequencing for quantitative analysis of CST6 methylation

Fresh-frozen tissues for pyrosequencing were available in only 51 IBC patients. The methylation status of *CST6 *gene in the fresh-frozen tissues was analyzed by pyrosequencing using a forward primer and a biotinylated reverse primer designed by PSQ Assay Design (Biotage AB, Uppsala, Sweden). The primer sequences for pyrosequencing were as follows: 5'-GGTTTTTTGGGTTTTTTGAA-3' (forward), 5'-Biotin TGAGGGTTTTGATGGTAT-3' (reverse) and 5'-TTGAATTTTGTAGGATTT-3' (sequencing primer). Briefly, 20 ng of sodium bisulfite-modified DNA was amplified in a 25 μl reaction with the primer sets and 5 U of Taq polymerase (Solgent Co, Daejeon, Korea). Samples were heated at 95°C for 10 minutes and then amplified for 40 cycles consisting of 95°C for 45 s, 55°C for 35 s and 72°C for 60 s. All reactions were then incubated at 72°C for 10 minutes and cooled to 4°C. The PCR products were visualized on a 1.5% agarose gel by ethidium bromide staining for verification. Pyrosequencing reactions were hot started at 94°C for 5 minutes, and amplification was carried out over 45 cycles (30 s at 94°C, 30 s at 58°C, 30 s at 72°C), followed by 5 minutes at 72°C with sequencing primers on the PSQ HS 96A System (Biotage AB, Uppsala, Sweden) according to the manufacturer's specifications. The methylation index for each CpG in the target region was calculated using the provided software.

### Statistical analysis

The normality of continuous variables was tested using the Shapiro-Wilk test. The Wilcoxon rank-sum test (or *t*-test) and Fisher's exact test (or the Chi-squared test) were used for the univariate analysis of the continuous and categorical variables, respectively. A multivariate logistic regression analysis was conducted to determine the relationship between cystatin M loss and any covariates found to be statistically significant in the univariate analysis, and to calculate odds ratios (ORs). Covariates with a *P*-value of <0.25 in the univariate analysis or any variables that were considered to be biologically important were subjected to the multivariate analysis. All statistical analyses were two-sided with a type I error rate of 5%.

## Results

### Clinicopathological characteristics of cystatin M loss

Heterogenous staining, defined in our study as when the staining pattern was different among cells within the same block, is often known to be seen in epigenetically silenced genes within advanced tumors. Heterogeneous staining was found in 4 (3%) of 117 DCISs and in 6 (3%) of 175 IBCs. When a slide showed heterogeneous staining, we grouped cells according to staining intensity and calculated the immunoreactive score (IS) of 0 to 7 for cystatin M expression in each group separately. We then calculated the mean IS in all groups. If the mean score was less than two, the tumor was considered to exhibit loss of cystatin M expression. The association between cystatin M loss and clinicopathological parameters in 117 DCISs and 175 IBCs is shown in Table 1. Cystatin M loss was found in 9 (8%) of the 117 DCISs and in 99 (56%) of 175 IBCs studied (*P *< 0.0001). The age of patients with DCIS or IBC was not significantly different according to cystatin M expression (*P *= 0.13 and *P *= 0.89, respectively). Cystatin M loss was found not to be associated with tumor size in patients with DCIS (*P *= 0.87) or IBC (*P *= 0.27). The mean tumor size was 2.9 cm and 2.6 cm for IBC patients with and without cystatin M loss, respectively. No association was found between cystatin M loss and the number of lymph nodes, family history of breast cancer in DCISs and IBCs. Furthermore, the histologic grade was not associated with cystatin M loss in DCISs (*P *= 0.90) or IBCs (*P *= 0.32). A statistically significant association was not found between cystatin M loss and pathologic stage in IBCs (*P *= 0.86).

**Table 1:** Clinicopathological characteristics

	DCIS			IBC		
		
	Cystatin M loss			Cystatin M loss		
				
	Yes (*n *= 9)	No (*n *= 108)	*P*-value	Yes (*n *= 99)	No (*n *= 76)	*P *-value
Age^1^	50 ± 9	46 ± 9	0.13	49 ± 11	49 ± 10	0.89
Tumor size (cm)^1^	2.7 ± 1.8	3.0 ± 2.1	0.87	2.9 ± 1.3	2.6 ± 1.1	0.27
Number of LN^1^	0	0.4 ± 1.1	0.69	2.6 ± 5.0	3.1 ± 5.9	0.83
Family history						
No	9	104		92	72	
Yes	0	4	1.00	7	4	0.76
Histologic grade						
I	1	24		8	8	
II	5	48		42	39	
III	3	36	0.90	49	29	0.32
ER						
Negative	1	32		50	24	
Positive	8	76	0.44	49	52	0.01
PR						
Negative	3	42		62	30	
Positive	6	66	1.00	37	46	0.002
HER2						
Negative	3	46		58	43	
Positive	6	62	0.73	41	33	0.79
HER4						
Negative	7	37		34	11	
Positive	2	71	0.03	65	65	0.003
Stage						
I				21	14	
II				55	43	
III				22	18	
IV				1	2	0.86

^1 ^Mean ± standard deviation.

DCIS, ductal carcinoma *in situ*; IBC, invasive breast cancer; ER, estrogen receptor; PR, progesterone receptor; LN, number of involved lymph node; Family history, a history of breast cancer within family.

### Relationship between cystatin M loss and the expression of ER, PR, HER2, and HER4

The prevalence of ER, PR, HER2, or HER4 loss was significantly different between DCISs and IBCs (Table 1). ER loss occurred in 33 (28%) of 117 DCISs and in 74 (42%) of 175 IBCs, and this difference was statistically significant (*P *= 0.01). The losses of PR and HER2 also occurred at a significantly different prevalence between DCISs and IBCs (*P *= 0.02 and *P *= 0.008, respectively). In addition, the prevalence of HER4 loss was 38% (44 of 117) and 26% (45 of 175) in DCISs and IBCs, respectively, and this difference was also statistically significant (*P *= 0.03). The relationship between cystatin M loss and the loss of ER, PR, HER2, or HER4 was analyzed in DCISs and IBCs (Table 1). Cystatin M loss was found in 1 (3%) of 33 ER-negative DCISs and in 8 (10%) of 84 ER-positive DCISs, and this difference was not statistically significant (*P *= 0.44). However, the expression status of ER in IBCs was significantly associated with cystatin M loss (*P *= 0.01). Cystatin M loss occurred in 50 (68%) of 74 ER-negative IBCs and in 49 (49%) of 101 ER-positive IBCs. The relationships between cystatin M loss and PR loss in DCISs and IBCs were similar to those between cystatin M loss and ER loss, but HER2 loss was not significantly associated with cystatin M loss in DCISs and IBCs (*P *= 0.73 and *P *= 0.79, respectively). Cystatin M loss was found in 34 (76%) of the 45 HER4-negative IBCs and in 65 (50%) of the 130 HER4-positive IBCs and this difference was statistically significant (*P *= 0.003).

**Figure 2:**
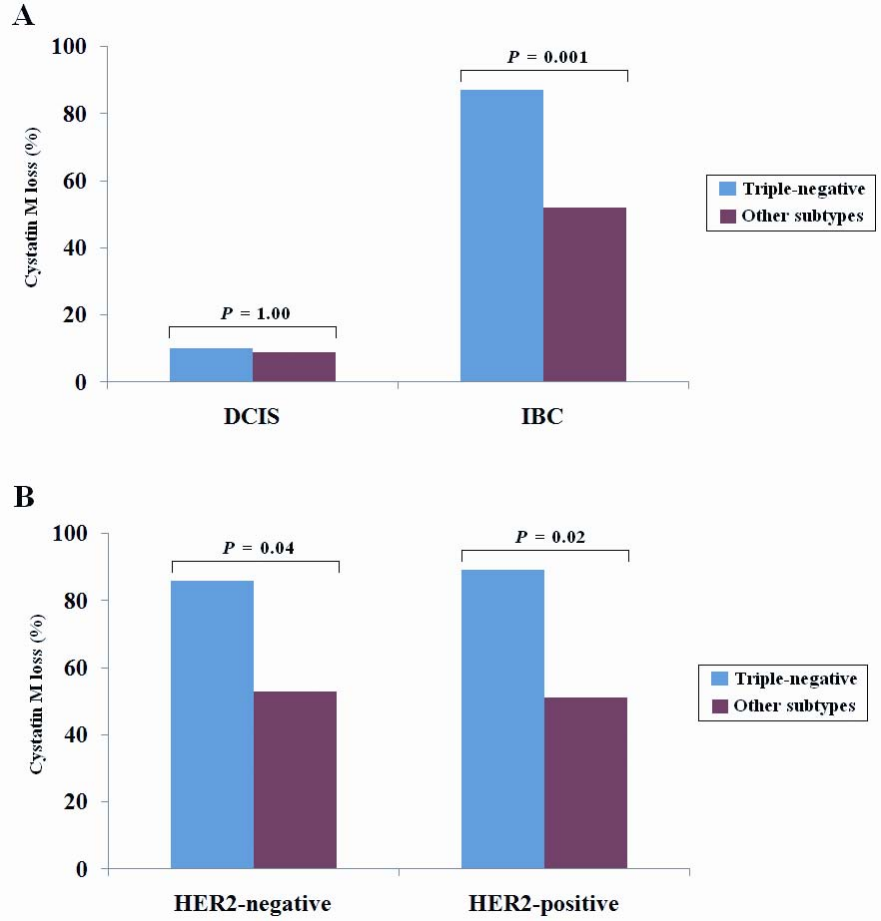
**Relationship between cystatin M loss and the triple-negative status of ER, PR, and HER4**. **(A) **Cystatin M loss occurred at a higher prevalence in the triple-negative IBCs than in other subtypes (*P *= 0.001). **(B) **The association between cystatin M loss and triple-negative IBCs was analyzed according to the expression status of HER2. For HER2-negative IBCs, cystatin M loss occurred in 12 (86%) of 14 triple-negative cases and in 46 (53%) of 87 other subtypes (*P *= 0.02). For HER2-positive IBCs, cystatin M loss was found in 8 (89%) of 9 triple-negative cases and in 33 (51%) of 65 other subtypes (*P *= 0.04). Triple-negative indicates IBCs with the losses of ER, PR, and HER4.

Triple-negative breast cancers (TNBCs) of ER, PR, and HER2, are known to be typically associated with poor prognosis due to aggressive behavior of the tumor and lack of targeted therapies. Therefore, we analyzed the association of triple-negative status of ER, PR, and HER2 with cystatin M loss. The triple-negative status of ER, PR, and HER2 occurred in 2 (2%) of 117 DCISs and in 35 (20%) of 175 IBCs. However, the triple-negative status was not associated with cystatin M loss in DCISs (*P *= 1.00) and IBCs (*P *= 0.08), and therefore the triple-negative status of ER, PR, and HER2 was not considered in the further analysis. Another triple-negative status of ER, PR, and HER4 occurred in 11 (9%) of 117 DCISs and in 23 (13%) of 175 IBCs. For the DCIS cases, cystatin M loss was not associated with the triple-negative status of ER, PR, and HER4 (*P *= 1.00). However, cystatin M loss in IBCs was significantly associated with the triple-negative status of ER, PR, and HER4 (*P *= 0.001; Figure 2A): cystatin M loss occurred in 20 (87%) of 23 triple-negative IBCs of ER, PR, and HER4 and in 79 (52%) of 152 other subtypes. To rule out the possibility of a confounding effect of HER2 in the relationship between cystatin M loss and the triple-negative status of ER, PR, and HER4, we stratified the data according to the expression status of HER2 and reanalyzed the relationship according to HER2. However, no confounding effect of HER2 was found in this study: cystatin M loss occurred at a higher prevalence in the triple-negative IBCs than in other subtypes irrespective of HER2 expression (Figure 2B).

### Logistic regression analysis of cystatin M loss

In univariate logistic regression analysis (Table 2A), cystatin M loss occurred at 2.20 times (95% confidence interval (CI) = 1.19 to 4.06; *P *= 0.02) higher prevalence in the ER-negative IBCs than in the ER-positive IBCs. Cystatin M loss also occurred at a high prevalence in the PR-negative IBCs (OR = 2.41, 95% CI = 1.32 to 4.39; *P *= 0.007) as well as in the HER4-negative IBCs (OR = 3.09, 95% CI = 1.44 to 6.62; *P *= 0.003) than in the PR- or HER4-positive cases, respectively. A multivariate logistic regression analysis was performed to control for the potential confounding effects of variables, such as age, and to calculate odds ratio (Table 2B). The coefficient for age variable in IBCs was not statistically significant in our univariate analysis (*P *= 0.89), but age was considered to be biologically important and, therefore, it was included in the multivariate analysis in order to better construct a parsimonious model. The losses of ER, PR, and HER4 were associated with each other (data not shown), and therefore the triple-negative status of ER, PR, and HER4 included in the multivariate logistic regression as a single covariate. Cystatin M loss occurred at a 3.57 times (95% CI = 1.28 to 9.98; *P *= 0.01) higher prevalence in the triple-negative IBCs than in other subtypes, after adjusting age. These observations suggest that the losses of ER, PR, and HER4 may be significantly associated with cystatin M loss in IBCs.

**Table 2:** Logistic regression analysis of the association between cystatin M loss and the losses of ER, PR, and HER4 in IBCs (N = 175)

	OR	95% CI	*P *-value
Univariate^1^			
ER-negative	2.20	1.19 to 4.06	0.02
PR-negative	2.41	1.32 to 4.39	0.007
HER4-negative	3.09	1.44 to 6.62	0.003
Multivariate^2^			
Triple-negative	3.57	1.28 to 9.98	0.01

^1 ^Reference was a group with expression of each protein. ^2 ^Age was controlled in multivariate analysis, and other subtypes with expression of either ER, PR, or HER4 was used as a reference. Triple-negative, the losses of ER, PR, and HER4; ER, estrogen receptor; PR, progesterone receptor; OR, odds ratio; CI, confidence interval.

### The association between *CST6 *methylation and the loss of cystatin M, ER, PR, or HER

**Figure 3:**
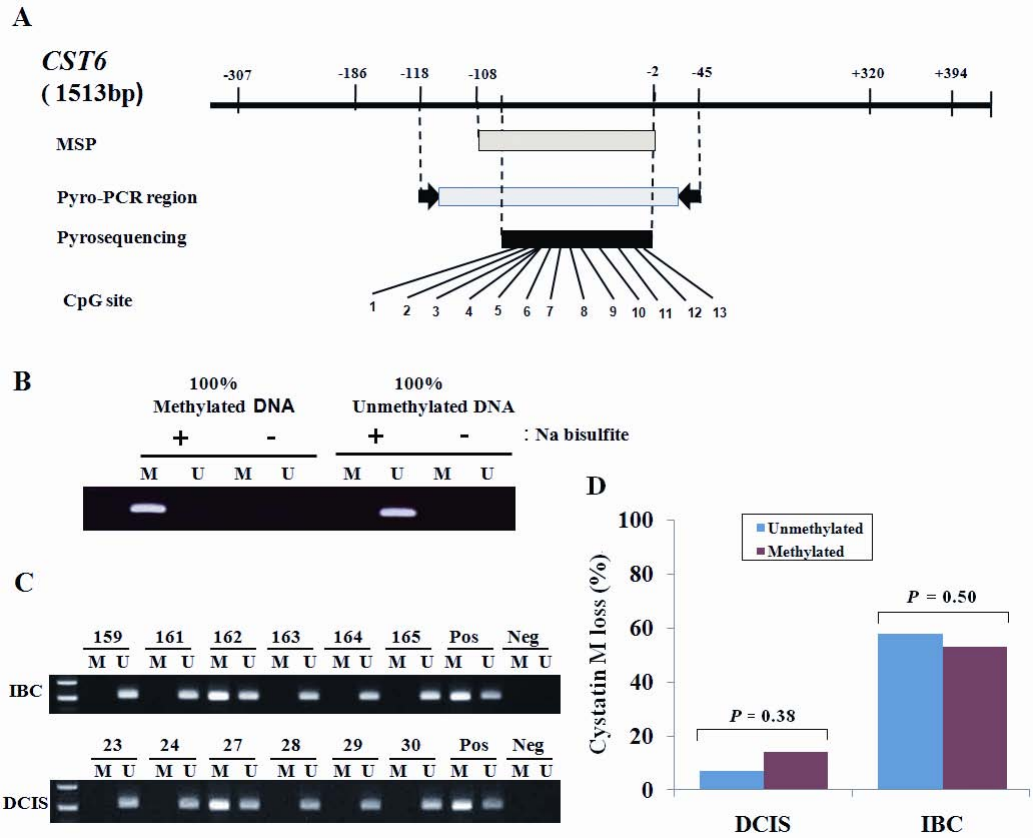
**Qualitative analysis of *CST6 *methylation by MSP**. **(A) **Locations of oligonucleotide primers for methylation-specific PCR (MSP) and pyrosequencing are shown relative to the transcriptional start site within the human *CST6 *gene. "Pyro-" represents pyrosequencing. **(B) **The sensitivity and specificity of the MSP primers were verified by amplifying 100% methylated DNA and 100% unmethylated DNA with sodium bisulfite treatment. **(C) **MSP for the *CST6 *gene was performed using methylation-specific (M) and unmethylation-specific (U) primer sets, respectively, in formalin-fixed paraffin-embedded tissues from IBC and DCIS. The numbers shown are sample identification numbers. "Pos" and "Neg" represents positive and negative controls for methylated (M) and unmethylated (U) allele, respectively. **(D) **No relationship was found between *CST6 *methylation by MSP and the cystatin M loss in DCIS (*P *= 0.38) and IBC (*P *= 0.50).

Epigenetic silencing of *CST6 *gene in breast cancer has been reported by several groups. To investigate whether the loss of ER, PR, or HER4 is associated with the hypermethylation of CpG island at the promoter region of the *CST6 *gene, we analyzed the methylation status of the *CST6 *gene using MSP. The primers designed for MSP yielded an amplicon spanning from nucleotides -108 to -2 (Figure 3A). Primers for MSP were validated using 100% methylated and 100% unmethylated DNA (Figure 3B). The hypermethylation of the *CST6 *gene by MSP was found in 22 (20%) of 108 DCISs studied and in 59 (34%) of 173 IBCs (Figure 3C). No association was also found between *CST6 *methylation and cystatin M loss in DCISs (*P *= 0.38) or IBCs (*P *= 0.50) (Figure 3D). The lack of association between cystatin M loss and hypermethylation of the *CST6 *gene may be due to the density dependence of methylated CpGs on transcriptional silencing by *CST6 *methylation or the inconsistence of methylation status between primer binding sites and other CpG sites within PCR product.

**Figure 4:**
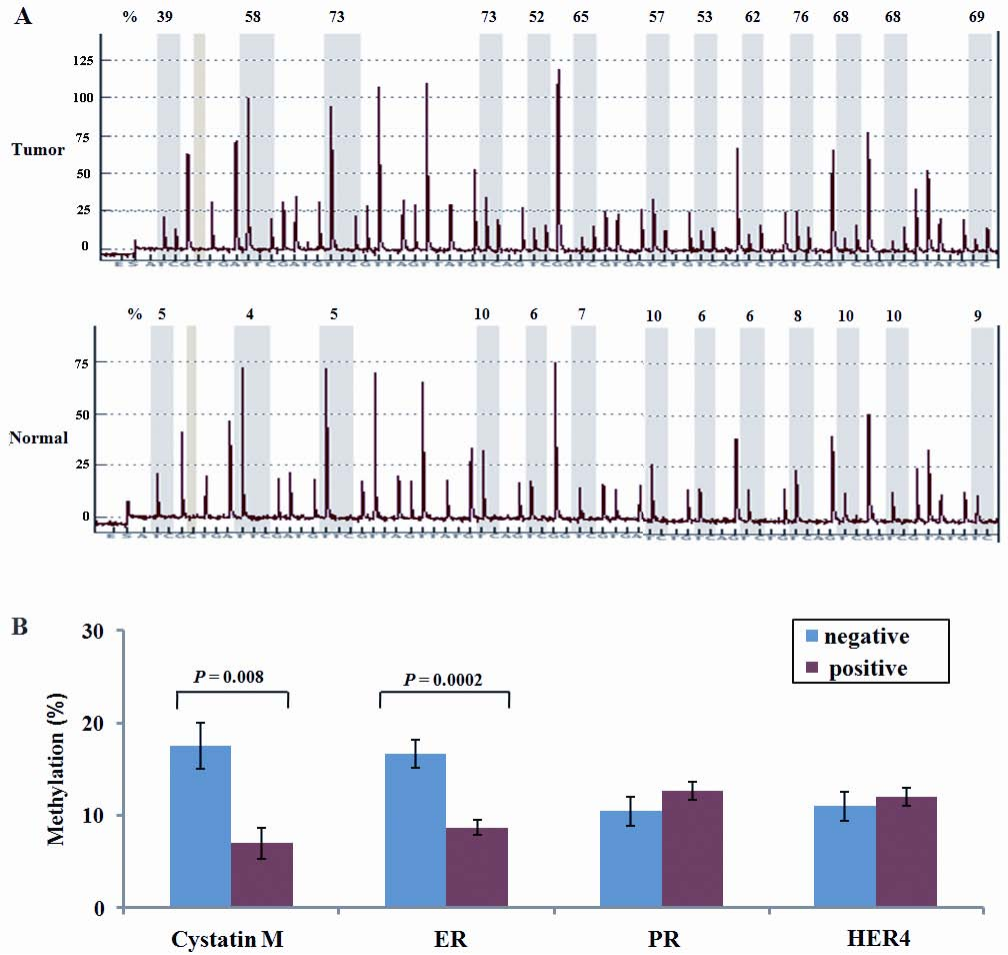
**Quantitative analysis of *CST6 *methylation by pyrosequencing**. **(A) **Representative examples of pyrosequencing are shown in tumor and matched normal tissues. Percent indicates the quantity of methylation at each CpG locus across all 13 CpG dinucleotides. **(B) **The quantities of methylated CpGs in IBC show a significant association with the cystatin M loss (*P *= 0.008) and ER loss (*P *= 0.0002), but not with the PR (*P *= 0.64) or HER4 loss (*P *= 0.87). The negative and positive indicate the presence and absence of expression of each protein, respectively. Error bars indicate a standard deviation.

The methylation status of 13 CpG sites (Figure 3A) in the region spanning from nucleotides -108 to -2 were, therefore, reassessed quantitatively using pyro-sequencing in 51 fresh-frozen tissues and matched normal tissues from IBC patients. The quantities of methylated CpGs between normal and tumor tissues were usually different (Figure 4A). The association between the quantity of *CST6 *methylation and the expression statuses of cystatin M, ER, PR, and HER4 in tumor tissues was analyzed (Figure 4B). The methylation level at each CpG in tumor tissues was adjusted by subtracting the quantity of methylated CpGs in matched normal tissue from that of methylated CpGs in tumor tissue. Average quantities of methylated CpGs in tumor tissues were significantly associated with cystatin M loss (*P *= 0.008; Wilcoxon rank sum test) or ER loss (*P *= 0.0002; Wilcoxon rank-sum test). The average quantities of methylated CpGs between groups with and without ER loss were 16.4% and 8.7%, respectively. However, the quantities of methylated CpGs were not associated with the expression status of PR (*P *= 0.64) or HER4 (*P *= 0.87).

## Discussion

Cystatin M is known to inhibit the activity of cystein proteases which degrade extracellular matrix components. In this study, cystatin M loss occurred in 9 (8%) of 117 patients with DCIS and in 99 (57%) of 175 patients with IBC, supporting previous reports [3,4,7] that cystatin M is one of many factors that are involved in the acquisition of an invasive cellular phenotype in breast cancer. In addition, cystatin M loss occurred more frequently in IBCs with the losses of ER, PR, and HER4 than without, suggesting that cystatin M expression may be influenced by synergistic effect of those proteins. However, the mechanism by which cystatin M expression is regulated by ER, PR, and HER4 awaits further elucidation. The expression statuses of ER, PR, and HER4 in this study were significantly associated with each other (data not shown) and PR isoforms are known to be expressed in response to ER or independently of ER [17]. In addition, molecules involved in cell adhesion to extracellular matix and cytoskeletal interaction are known to be regulated by induced PR even in the absence of ligand [18,19]. Based on these observations, one possibility is that cystatin M may be a downstream target of HER4-ER in IBC and be influenced by PR.

The HER2 is well known as a major player in initiation or progression of breast cancer, but the significance of HER4 in breast cancer has not been studied extensively. Triple-negative status of ER, PR, and HER2 was not associated with cystatin M loss, but another triple-negative status of ER, PR, and HER4 was significantly associated with cystatin M loss in IBC, suggesting a different role of HER4 independently of HER2. Although the interaction of HER4 with PR is not known in breast cancer, a positive association between HER4 and ER has been reported by many groups. HER4 overexpression in ER-positive breast cancer cells results in enhanced cell growth and estrogen response element (ERE)-mediated transcriptional activity, and ectopically expressed as well as endogenous HER4 interacts with ligand-bound ER in response to estrogen and potentiates ER transactivation [13,14]. Suo *et al*. [11] found that MCF-7 and T47-D breast cancer cells responsive to hormonal therapy were ER- and HER4-positive and that MDA-MD-231 and SK-BR-3 cells nonresponsive to hormonal therapy were ER- and HER4-negative. In addition, clinical observations have reported that the co-expression of ER and HER4 in breast cancer is associated with a prognostically favorable outcome [10,12,13]. The present study also found that ER was associated with HER4 in IBCs (*P *= 0.01). These observations suggest that HER4 and ER may co-operate functionally in breast cancer.

What is then the role of HER4 in the interaction with ER in breast cancer? It has been reported that HER4 is an estrogen-target gene, which is inducible upon E2 stimulation by recruiting ER to the HER4 promoter. Once HER4 is activated by its own ligands, such as heregulin, HER4 is processed by TNFα-converting enzyme (TACE) followed by γ-secretase, which results in the release of the ectodomain fragment and soluble intracellular domain (4ICD) [20-22]. HER4 ICD has two isoforms by alternative pre-mRNA splicing and various motifs for association with signaling molecules, such as PI3K and Yes-associated protein (YAP) [23,24]. HER4/4ICD possesses constitutively active kinase activity and is a chaperone for nuclear entry of signal transducer and activator of transcription 5A (STAT5A). Accordingly, HER4/4ICD forms a complex with ER and is translocated into the nucleus upon estrogen stimulus, and the nuclear ER/4ICD complex co-activates ER transcription by being selectively recruited to estrogen responsive gene promoters such as progesterone receptor (*PgR*) [14,21]. These preclinical studies indicate that HER4 contributes to ER translocation to target genes and functions as an ER transcriptional co-regulator, selectively binding with ER to gene promoters harboring ERE. However, it is unclear if HER4 functions as a co-regulator of ER in the absence of ERE.

To the best of our knowledge, cystatin M has not previously been suggested as a downstream target of ER in breast cancer. We searched for putative ER target sequences within the promoter of the *CST6 *by using the motif search program [25]. While the *CST6 *promoter does not contain any ERE or half-ERE sites for ER binding, it does contain binding sites for other transcription factors such as SP-1 or AP-1. ER is also known to interact with other transcription factors such as AP-1 and SP-1 [26,27]. These observations suggest that the effect of ER on cystatin M expression may not be mediated directly through ER-binding to the promoter of the *CST6 *gene. Further work will be necessary to understand possible mechanisms underlying the cystatin M loss by ER in breast cancer.

## Conclusions

The biological meaning of the cutoff criteria for positive expression that was adopted in this study was not clear, and poorly justified cutoffs may lead to wrong conclusions and contribute to non-reproducibility of results. The cutoff value used to define positive expression of a protein in immunohistochemistry is of critical importance, and a more optimal threshold should be developed for non-biased conclusions. For the quantitative analysis of methylation status in IBC, this study was severely limited by the small number of fresh-frozen tissues (51 samples) which may have led to an incorrect conclusion between ER expression and *CST6 *methylation. Furthermore, patient prognosis according to cystatin M loss was not analyzed due to a short period of follow-up. Accordingly, additional work in a large number of samples will also be required to precisely determine the role of cystatin M as a prognostic marker and to understand the association of epigenetic modification with cystatin M loss. Taken together, the present study suggests that cystatin M loss in IBC may be associated with triple negative status of ER, PR, and HER4.

## Abbreviations

DCIS: ductal carcinoma *in situ*; ER: estrogen receptor; ERE: estrogen response element; IBC: invasive breast cancer; ICD: intracellular domain; IS: immunoreactive score; MSP: methylation-specific; PBS: phosphate-buffered saline; PCR: polymerase chain reaction; PR: progesterone receptor; SDF-1: stromal cell-derived factor 1; STAT5A: signal transducer and activator of transcription 5A; TACE: TNFα-converting enzyme; TMA: tissue microarray; TNBCs. Triple-negative breast cancers; TNM: tumor-node-metastasis; YAP: Yes-associated protein.

## Competing interests

The authors declare that they have no competing interests.

## Authors' contributions

EK, SEP, YK, and JAH carried out the experiments and some data analysis. YSL, SJN, SB, JP and DHK participated in study design and data interpretation. EK, SEP, and YK conducted sample collection and immunohistochemistry under the supervision of EYC. EK, SEP and DHK drafted the manuscript. All authors had final approval of the manuscript.
